# Does prolonged television viewing affect Body Mass Index? A case of the Kingdom of Saudi Arabia

**DOI:** 10.1371/journal.pone.0228321

**Published:** 2020-01-30

**Authors:** Mohammed Khaled Al-Hanawi, Gowokani Chijere Chirwa, Laeticia Amiss Pemba, Ameerah M. N. Qattan

**Affiliations:** 1 Department of Health Services and Hospital Administration, Faculty of Economics and Administration, King Abdulaziz University, Jeddah, Saudi Arabia; 2 Centre for Health Economics, University of York, Heslington, York, United Kingdom; 3 Economics Department, Chancellor College, University of Malawi, Zomba, Malawi; University of Botswana, BOTSWANA

## Abstract

**Background:**

The rising prevalence of overweight and obesity and their health implications is a major public health concern worldwide. This study set out to understand the relationship between the number of hours spent watching television and Body Mass Index (BMI) in the Kingdom of Saudi Arabia using data from the Saudi Health Interview Survey.

**Methods:**

The study employed both ordinary least squares and quantile regressions to estimate the mean and distributional association of prolonged television watching and BMI.

**Results:**

The findings showed that prolonged television viewing is associated with larger BMI values. Additionally, the relationship was found to be greater towards the lower and upper tails of the BMI range and insignificant in the middle of the BMI distribution. Furthermore, the findings also showed that there is a gender gap in BMI levels, where females are likely to have higher BMI values than males.

**Conclusions:**

The creation of more proactive recreational programs that can act as substitutes to television watching is recommended in order to reduce the amount of time that individuals spend watching television. It is also recommended that such interventions are tailored towards improving females’ levels of physical activity. The inclusion of television programs aimed at encouraging physical exercise and healthy diets is also imperative.

## Introduction

The rising prevalence of overweight and obesity and their health implications is a major public health concern worldwide [1, 2]. This accelerated prevalence of overweight and obesity worldwide is due to changes in diet and increasing physical inactivity. Recent estimates found that roughly 500 million adults worldwide are obese [3]. Furthermore, it is well known that morbidity and mortality rates increase as body weight increases. According to the World Health Organization (WHO), obesity is one of the top leading global risks for mortality worldwide [4]. It is one of the major causes of chronic diseases, and it has been reported that dietary risks account for more deaths worldwide than tobacco and alcohol combined [5]. Overweight and obesity have some profound health costs. They contribute to roughly 4 million deaths (7.1% of all deaths) and 120 million healthy years of life lost (disability-adjusted life years or DALYs). Worldwide, this culminates to 4.9% of all DALYs among the adult population [6].

The prevalence of obesity is higher in Arabian Gulf countries including the Kingdom of Saudi Arabia (KSA) than most other countries in the world [7]. Indeed, the KSA has witnessed an increase in cases of non-communicable diseases (NCDs) such as obesity and diabetes, and it has one of the highest rates of overweight and obesity worldwide [8–10]. While potential contributors to obesity are multiple and complex, prolonged television viewing has been cited as one of the main risk factors that contribute to an increase in the risk of NCDs, including obesity [11–15]. Several studies have proposed possible mechanisms for this, including the displacement of physical activity, increasing sleep deprivation, increased calorie intake while watching television, and consequent use of foods commonly advertised on television [16, 17]. Moreover, positive associations between spending a long time watching television and higher body mass index (BMI) values have been shown [18, 19].

BMI is a simple weight-for-height index that is usually used to classify adults as underweight, overweight, and obese. It is calculated as an individual’s weight in kilograms divided by the square of their height in meters (kg/m^2^) [20]. According to the BMI weight status categories, anyone with a BMI below 18.5 is classified as underweight, anyone with a BMI between 18.5 and below 25 is classified as having a normal weight, anyone between 25 and 29.9 is classified as overweight, and anyone with a BMI over 30 is classified as obese [21].

Increased television viewing time is associated with an increase in sitting time and the intake of energy dense foods, which results in higher BMI levels [13, 22, 23]. Evidence has shown that greater rates of television viewing are directly associated with a higher risk of being overweight or obese, a higher BMI, and increased adiposity [24]. For instance, spending two hours watching television daily increases the relative risks of developing type 2 diabetes, cardiovascular disease, and all-cause mortality by 13%–20% [25].

While several studies have investigated the impact of prolonged television viewing on BMI [22, 26], no previous research has investigated the impact of prolonged television viewing on BMI in the KSA. Existing evidence has been drawn from Western developed countries such as United States of America, United Kingdom, Canada, among others, whose definition of obesity differs from that of Asian countries [27, 28] and may have a different impact on the television–BMI relationship. Furthermore, the number of hours spent watching television might be affected by the state strict programme controls in countries like KSA than most western countries. This highlights the need to investigate this issue in the KSA context in which obesity and overweight are serious concerns. Therefore, this study set out to understand the relationship between prolonged television viewing and BMI in the KSA. To the best of the authors’ knowledge, this study is the first to examine the association between television viewing and BMI in the KSA using nationally representative data. This study fills a gap in the literature and contributes to knowledge in this area. In particular, this study contributes to the existing literature by using quantile regressions to assess the relationship throughout the distribution. Unlike previous studies, the study uses household health survey data for KSA—which is one of the countries experiencing a huge BMI transition.

The KSA provides an intuitive case for the current study, given that for a long period it has been a closed country whose health dynamics remain partially studied. In addition, existing evidence shows that there has been a huge rise in obesity in the KSA. By the year 2016, the prevalence of obesity was around 35% in the population of adults aged over 18. The prevalence of obesity was lower (17%) in adolescents (age 10–19 years) [29]. However, for such a young population, the prevalence still remains worrisome. Furthermore, the methods used in the study are different and quite rigorous given that the estimations go beyond the mean, to examine the outcomes across the distribution. Estimation beyond the mean is important for giving direction on specific points at which policies can focus [30–32].

## Materials and methods

### Data and sample

The study used data from the Saudi Health Interview Survey (SHIS). This was a national survey carried out collaboratively by the Saudi Ministry of Health, the Institute for Health Metrics and Evaluation (IHME), and the University of Washington. The SHIS collected data on health and demographic characteristics primarily to assess the prevalence of several chronic conditions and to identify their risk factors. A multistage stratified probability sample was employed by the SHIS to recruit the respondents. A total of 10,735 individuals aged over 15 were interviewed out of 12,000 households originally contacted, giving a response rate of about 90%. Detailed descriptions of the sampling methodology and data collection of the SHIS are available elsewhere [33, 34]. In this study, the analysis was restricted to only those variables with no missing observations among those who were randomly sampled to have their weight taken in the data; as such, a total of 7746 observations were analyzed.

### Variables

Our outcome variable was BMI, which was computed from an anthropometry module that included data on weight and height, among others. BMI is calculated from the height and weight of an individual, that is to say, the ratio of the weight in kilograms divided by the square of the height in meters. All other covariates were based on previous empirical studies that have shown evidence of factors that can affect the BMI [22, 26, 35–38]. In the analysis, we used the natural log of BMI.

The main independent variable was the number of hours spent watching television per week. This was captured as a continuous variable and entered into the equation as a natural log. Socioeconomic status is controlled by income. The SHIS collected the grouped monthly income (in Saudi Riyal (SR)), and this was grouped into six categories: less than 3000 SR; 3000 to less than 5000 SR; 5000 to less than 7000 SR; 7000 to less than 10,000 SR; 10,000 to less than 15,000 SR; and 15,000 SR or more. The income variables were entered as a binary taking a value of 1 if a condition held and zero otherwise. The group with less than 3000 SR was used as the reference category.

We also controlled for education using the following groups: below primary (reference), primary school, intermediate school, high school, and higher education. Similarly, the age variable was entered in groups: 15–24 (reference category), 25–34, 35–44, 45–54, and 55 or above. Gender was coded as one if male and zero if female. Marital status was also captured as a binary, where a value of one was used if married and zero was used otherwise. We also controlled for the thirteen administrative regions, namely Almadina Almonawra, Albaha, Aljouf/Quriat, Aseer/Bisha, Eastern Region, Haiel, Jazan, Najran, Northern Borders, Qaseem, Riyadh,Tabouk, and the Western Region.

### Data analysis

In this study, the main econometric method used was the Quantile Regression (QR) method that was developed by Koenker and Bassett [39]. The QR framework enabled us to check whether the association of the covariates with BMI changed across the distribution of the BMI. Following Buchinsky [40] and Costa-Font, Fabbri, Gil [41], we let *Q_θ_*(*w*|*X*) for *θ*∈(0,1) denote the *θth* conditional quantile of the distribution of (log) BMI(*w*) given a vector *X* of *k* covariates. Thus, the conditional quantiles were expressed as

$$Q_{\theta }(w|X)=X^{\prime }\beta (\theta ),$$ (1)

where *β*(*θ*) is a vector of coefficients, namely, the QR coefficients and X′ is a vector of coefficients. Using the linear programming method, Eq (1) was then estimated by minimizing the objective function in Eq (2) with respect to *β*:

$$n^{-1}[\sum_{i:w_{i}\geq X_{i}^{\prime }\;\beta }^{n}\rho (w_{i}-X_{i}^{\prime }\;\beta )+\sum_{i:w_{i}<X_{i}^{\prime }\;\beta }^{n}\left(1-\rho )(w_{i}-X_{i}^{\prime }\beta \right)]\;{\forall i=1..,n;Q}_{\theta }(\varepsilon |X).$$ (2)

Empirically, the minimized Eq in (2) can thus be expressed as

$$Q_{BMI,\theta }=\beta_{0}+\beta_{1}(\theta )\;{TV}_{i}+X_{i}^{\prime }\;\beta_{i}(\theta )+\varepsilon_{i},$$ (3)

where *θ* is the quantile, and *Q_BMI,θ_* is the BMI of an individual *i* at that quantile. The variable *TV_i_* refers to the number of hours an individual spends on television; thus, *β*_1_ shows the association of television viewing and BMI. The controls were captured by the vector $X_{i}^{\prime }$, and *ε_i_* captured the unobserved factor, which can also affect BMI. We used bootstrapping with 500 replications in order to have the appropriate number of standard errors [39]. We also carried out an ordinary least-squares (OLS) analysis to check the robustness of the results. All analyses were carried out using Stata/MP Version 15.1.

### Ethical consideration

In this study, we have used records on age, weight, height, education, socioeconomic status, diet behaviour and watching of television. We did not use any information which involved the collection of human tissue. The study protocol was approved by the Saudi Ministry of Health and its Institutional Review Board (IRB). The participants consented and agreed to participate in the study. They were informed that participation is voluntary and they could stop at any time. Furthermore, they were also informed that the data would be used for the purpose of research at a future time point. After all the data was collected, it was then fully anonymised by the Ministry of Health before we accessed it. Thus, it is not possible to identify people using current data.

## Results

The results start with the presentation of the descriptive statistics showing the social and demographic characteristics in Table 1. At the time of the survey, the mean BMI was 28.51(Kg/m^2^), and minimum was 12 (Kg/m^2^), whereas the maximum was 223 (Kg/m^2^). On average, people spent about 25 hours per week watching television. Most of the respondents were married (67%), and 53% were males. The monthly income for around 72% of the respondents was less than 10,000 SR, while only 12% earned 15,000 SR or more. In terms of education, about 18% of the respondents were educated at below primary school level, while 26% of the respondents had completed college/university or a post-graduate degree. On average, the respondents ate red meat 3 days a week, whereas soda (including Coca-Cola, among others) was consumed 4 days per week.

**Table 1:** Socioeconomic and demographic characteristics.

Explanatory variable	Mean	*N*	Med	Min	Max
Body Mass Index (BMI)	28.51	7746	27	12	223
Hours spent watching television	25.14	7746	19	2	71
Married	67.00%	5190	1	0	1
Male	53.00%	4105	1	0	1
Education					
Below primary school	18.00%	1394	0	0	1
Primary school	10.00%	775	0	0	1
Intermediate school	29.00%	2246	0	0	1
High school	17.00%	1317	0	0	1
College/University +	26.00%	2014	0	0	1
Employed	65.00%	5035	1	0	1
Age					
15–24	20.59%	1595	0	0	1
25–34	26.74%	2071	0	0	1
35–44	22.94%	1777	0	0	1
45–54	14.03%	1087	0	0	1
≥55	15.70%	1216	0	0	1
Monthly income					
<3000 SR	17.43%	1350	0	0	1
3000 to less than 5000 SR	18.58%	1439	0	0	1
5000 to less than 7000 SR	16.58%	1284	0	0	1
7000 to less than 10,000 SR	19.12%	1481	0	0	1
10,000 to less than 15,000 SR	15.98%	1238	0	0	1
≥15,000 SR	12.31%	954	0	0	1
No. of days that soda is consumed	3.98	7746	3	1	7
No. of days that red meat is consumed	3.18	7746	3	1	7
Region					
Almadina Almonawra	6.38%	494	0	0	1
Albaha	7.23%	560	0	0	1
Aljouf/Quriat	4.14%	321	0	0	1
Aseer/Bisha	9.11%	706	0	0	1
Eastern Region	7.05%	546	0	0	1
Haiel	6.83%	529	0	0	1
Jazan	7.69%	596	0	0	1
Najran	7.68%	595	0	0	1
Northern Borders	5.69%	441	0	0	1
Qaseem	3.42%	265	0	0	1
Riyadh	13.93%	1079	0	0	1
Tabouk	4.96%	384	0	0	1
Western Region	15.88%	1,230	0	0	1
***N*** = 7746					

Table 2 shows the results of the OLS estimation of the association between the number of hours spent watching television and the BMI. In Table 2, column (1) shows the coefficients from the OLS regression, and column (2) shows the 95% confidence intervals (CI). Given that BMI and television hours are all expressed in log values, this means that the results can then be interpreted as partially elastic [42, 43]. The television coefficient of 0.010 (p < 0.01), means that a 1% increase in the number of hours spent watching television is associated with a 0.01% increase in the BMI, holding all other things constant.

**Table 2:** Regression results for the association of television viewing and BMI (OLS).

	(1)	(2)
Explanatory variable	logBMI	95% CI
Television hours	0.010***	[0.004, 0.015]
Married	0.021***	[0.007, 0.035]
Male	-0.046***	[-0.058, -0.035]
Education		
Primary school	-0.024**	[-0.045, -0.003]
Intermediate school	-0.011	[-0.031, 0.008]
High school	-0.021**	[-0.041, -0.000]
College/University +	-0.025**	[-0.044, -0.005]
Employed	0.029***	[0.015, 0.042]
Age		
25–34	0.087***	[0.065, 0.109]
35–44	0.148***	[0.125, 0.170]
45–54	0.174***	[0.150, 0.199]
≥55	0.164***	[0.140, 0.188]
Monthly income		
3000 to less than 5000 SR	0.022**	[0.002, 0.042]
5000 to less than 7000 SR	0.024**	[0.004, 0.044]
7000 to less than 10,000 SR	0.014	[-0.007, 0.035]
10,000 to less than 15,000 SR	0.023**	[0.001, 0.044]
≥15,000 SR	0.022*	[-0.002, 0.047]
No. of days that soda is consumed	0.002	[-0.005, 0.009]
No. of days that red meat is consumed	0.009*	[-0.002, 0.020]
Region		
Almadina Almonawra	-0.040***	[-0.066, -0.015]
Albaha	-0.033***	[-0.056, -0.010]
Aljouf/Quriat	0.016	[-0.017, 0.048]
Aseer/Bisha	-0.045***	[-0.068, -0.023]
Eastern Region	-0.019	[-0.043, 0.005]
Haiel	-0.002	[-0.026, 0.021]
Jazan	-0.091***	[-0.116, -0.066]
Najran	-0.034***	[-0.060, -0.009]
Northern Borders	-0.006	[-0.038, 0.026]
Qaseem	-0.010	[-0.041, 0.022]
Tabouk	0.020	[-0.013, 0.053]
Western Region	-0.045***	[-0.065, -0.025]
_cons	3.183***	[3.148, 3.218]
***N*** = 7746		

95% confidence intervals in brackets

* p < 0.10

** p < 0.05

*** p < 0.01

Also of interest is the gender variable coefficient. The negative sign indicates that males are likely to have a lower BMI than females by at least 0.046 (p < 0.01). Furthermore, for all age groups, the BMI is higher than that of the reference category of people aged 24 years or below. Whilst the income variables suggest that BMI is significantly higher for those within the upper income categories than the people earning 3000 SR or less, there is no difference compared with individuals earning in the 7000 to less than 10,000 SR category. Given that the OLS cannot show heterogeneity across quantiles, we then carried out a quantile regression analysis. The next table (Table 3) shows the results for the quantile regression.

**Table 3:** Regression results for the association of television viewing and BMI (Quantile).

Explanatory variable	Q10	Q25	Q50	Q75	Q90
Television hours	0.012***	0.006**	0.005	0.005	0.013**
	[0.005, 0.020]	[0.000, 0.012]	[-0.001, 0.011]	[-0.002, 0.012]	[0.003, 0.024]
Married	0.042***	0.032***	0.029***	0.015	-0.010
	[0.022, 0.062]	[0.017, 0.048]	[0.015, 0.044]	[-0.003, 0.032]	[-0.036, 0.016]
Male	-0.021**	-0.041***	-0.050***	-0.059***	-0.067***
	[-0.036, -0.005]	[-0.053, -0.029]	[-0.062, -0.039]	[-0.073, -0.045]	[-0.087, -0.047]
Education					
Primary school	-0.016	-0.022*	-0.053***	-0.025*	-0.023
	[-0.046, 0.015]	[-0.046, 0.001]	[-0.075, -0.031]	[-0.052, 0.002]	[-0.062, 0.017]
Intermediate school	0.019	-0.012	-0.029***	-0.030**	-0.017
	[-0.009, 0.048]	[-0.034, 0.010]	[-0.050, -0.009]	[-0.055, -0.005]	[-0.053, 0.020]
High school	-0.011	-0.015	-0.035***	-0.034**	-0.013
	[-0.041, 0.019]	[-0.038, 0.008]	[-0.057, -0.014]	[-0.061, -0.008]	[-0.051, 0.025]
College/University +	0.010	-0.022*	-0.042***	-0.045***	-0.031
	[-0.019, 0.040]	[-0.045, 0.001]	[-0.063, -0.020]	[-0.072, -0.019]	[-0.068, 0.007]
Employed	0.047***	0.042***	0.034***	0.017*	0.020
	[0.027,0.067]	[0.026,0.057]	[0.020,0.049]	[-0.000,0.035]	[-0.005,0.045]
Age					
25–34	0.071***	0.114***	0.110***	0.105***	0.083***
	[0.043, 0.099]	[0.092, 0.135]	[0.090, 0.130]	[0.080, 0.129]	[0.047, 0.119]
35–44	0.147***	0.178***	0.161***	0.167***	0.144***
	[0.117, 0.178]	[0.154, 0.202]	[0.139, 0.183]	[0.140, 0.194]	[0.105, 0.183]
45–54	0.182***	0.207***	0.186***	0.179***	0.145***
	[0.149, 0.215]	[0.181, 0.233]	[0.162, 0.210]	[0.150, 0.209]	[0.102, 0.187]
≥55	0.201***	0.208***	0.188***	0.161***	0.132***
	[0.167, 0.234]	[0.182, 0.234]	[0.164, 0.212]	[0.131, 0.191]	[0.089, 0.175]
Monthly income					
3000 to less than 5000 SR	0.037***	0.032***	0.028***	0.009	0.023
	[0.012, 0.063]	[0.012, 0.052]	[0.010, 0.046]	[-0.013, 0.032]	[-0.010, 0.055]
5000 to less than 7000 SR	0.061***	0.057***	0.035***	0.016	0.017
	[0.034, 0.087]	[0.037, 0.078]	[0.015, 0.054]	[-0.008, 0.040]	[-0.018, 0.051]
7000 to less than 10,000 SR	0.059***	0.047***	0.024**	-0.009	-0.011
	[0.032, 0.086]	[0.026, 0.068]	[0.004, 0.043]	[-0.033, 0.015]	[-0.045, 0.024]
10,000 to less than 15,000 SR	0.067***	0.055***	0.031***	-0.003	-0.012
	[0.038, 0.095]	[0.033, 0.077]	[0.011, 0.052]	[-0.029, 0.022]	[-0.049, 0.025]
≥15,000 SR	0.060***	0.056***	0.038***	0.004	-0.021
	[0.029, 0.090]	[0.032, 0.080]	[0.016, 0.061]	[-0.023, 0.031]	[-0.060, 0.018]
No. of days that soda is consumed	-0.010**	-0.003	-0.002	0.008	0.007
	[-0.020, -0.000]	[-0.011, 0.005]	[-0.009, 0.006]	[-0.002, 0.017]	[-0.006, 0.021]
No. of days that red meat is consumed	0.017**	0.010*	0.006	0.009	-0.008
	[0.003, 0.032]	[-0.001, 0.022]	[-0.005, 0.016]	[-0.004, 0.022]	[-0.027, 0.010]
Region					
Almadina Almonawra	-0.058***	-0.033**	-0.032**	-0.038**	-0.013
	[-0.094, -0.022]	[-0.061, -0.006]	[-0.058, -0.006]	[-0.070, -0.007]	[-0.058, 0.033]
Albaha	-0.015	-0.007	-0.022*	-0.024	-0.008
	[-0.050, 0.019]	[-0.034, 0.020]	[-0.047, 0.003]	[-0.054, 0.007]	[-0.052, 0.036]
Aljouf/Quriat	0.013	0.012	0.001	0.023	0.063**
	[-0.029, 0.054]	[-0.020, 0.044]	[-0.029, 0.031]	[-0.014, 0.060]	[0.009, 0.116]
Aseer/Bisha	-0.042***	-0.049***	-0.050***	-0.039***	0.009
	[-0.074, -0.010]	[-0.074, -0.024]	[-0.073, -0.026]	[-0.067, -0.010]	[-0.032, 0.049]
Eastern Region	-0.022	-0.009	-0.014	-0.022	-0.016
	[-0.056, 0.012]	[-0.035, 0.018]	[-0.038, 0.011]	[-0.053, 0.008]	[-0.060, 0.028]
Haiel	0.009	0.022	0.006	-0.001	-0.005
	[-0.026, 0.043]	[-0.005, 0.049]	[-0.019, 0.031]	[-0.031, 0.030]	[-0.049, 0.040]
Jazan	-0.091***	-0.081***	-0.091***	-0.075***	-0.073***
	[-0.125, -0.057]	[-0.107, -0.055]	[-0.116, -0.067]	[-0.105, -0.045]	[-0.116, -0.030]
Najran	-0.021	-0.019	-0.029**	-0.015	-0.026
	[-0.055, 0.013]	[-0.045, 0.007]	[-0.053, -0.004]	[-0.045, 0.014]	[-0.069, 0.017]
Northern Borders	-0.027	-0.022	-0.021	-0.011	0.017
	[-0.064, 0.010]	[-0.050, 0.007]	[-0.048, 0.006]	[-0.043, 0.022]	[-0.030, 0.065]
Qaseem	-0.028	-0.005	0.015	0.007	0.026
	[-0.073, 0.017]	[-0.039, 0.030]	[-0.017, 0.048]	[-0.032, 0.047]	[-0.031, 0.084]
Tabouk	0.001	0.022	-0.011	0.026	0.012
	[-0.038, 0.040]	[-0.008, 0.053]	[-0.039, 0.017]	[-0.009, 0.060]	[-0.038, 0.062]
Western Region	-0.065***	-0.045***	-0.040***	-0.025**	0.017
	[-0.093, -0.038]	[-0.066, -0.024]	[-0.060, -0.020]	[-0.049, -0.001]	[-0.018, 0.052]
_cons	2.837***	2.983***	3.174***	3.338***	3.481***
	[2.791, 2.884]	[2.947, 3.019]	[3.140, 3.207]	[3.296, 3.379]	[3.421, 3.541]
**N**	7746	7746	7746	7746	7746

95% confidence intervals in brackets

* p < 0.10

** p < 0.05

*** p < 0.01

Our regression output was done for the 10^th^ percentile (Q10), 25^th^ percentile (Q25), median (Q50), 75^th^ percentile (Q75), and the 90^th^ percentile (Q90). Whilst the coefficient for the association of prolonged television watching and BMI is constant in the OLS models presented in Table 2, the results in Table 3 show that it differs among the quantile regression models estimated. Thus, this supports the notion that mean based regression misses out some important information in the entire distribution of the variables of interest. The significance and size of the coefficients differ by the specific quantile observed. In the 25^th^ percentile, there is a strong relationship between the number of hours spent watching television and the BMI (0.006; p < 0.05). However, the association is reduced in the 50^th^ and 75^th^ percentiles and becomes significant again in the 90^th^ quantile (0.013; p < 0.05). As the case of the OLS, of interest is also the gender coefficient. It is shown that the gender differential in BMI increases with an increase in the quantile. However, it is consistently negative, indicating that males are likely to have lower BMI values throughout the distribution. Furthermore, BMI increases with an increase in age; it is much higher for people in higher age groups than for people in lower age groups, for all quantiles.

**Fig 1:**
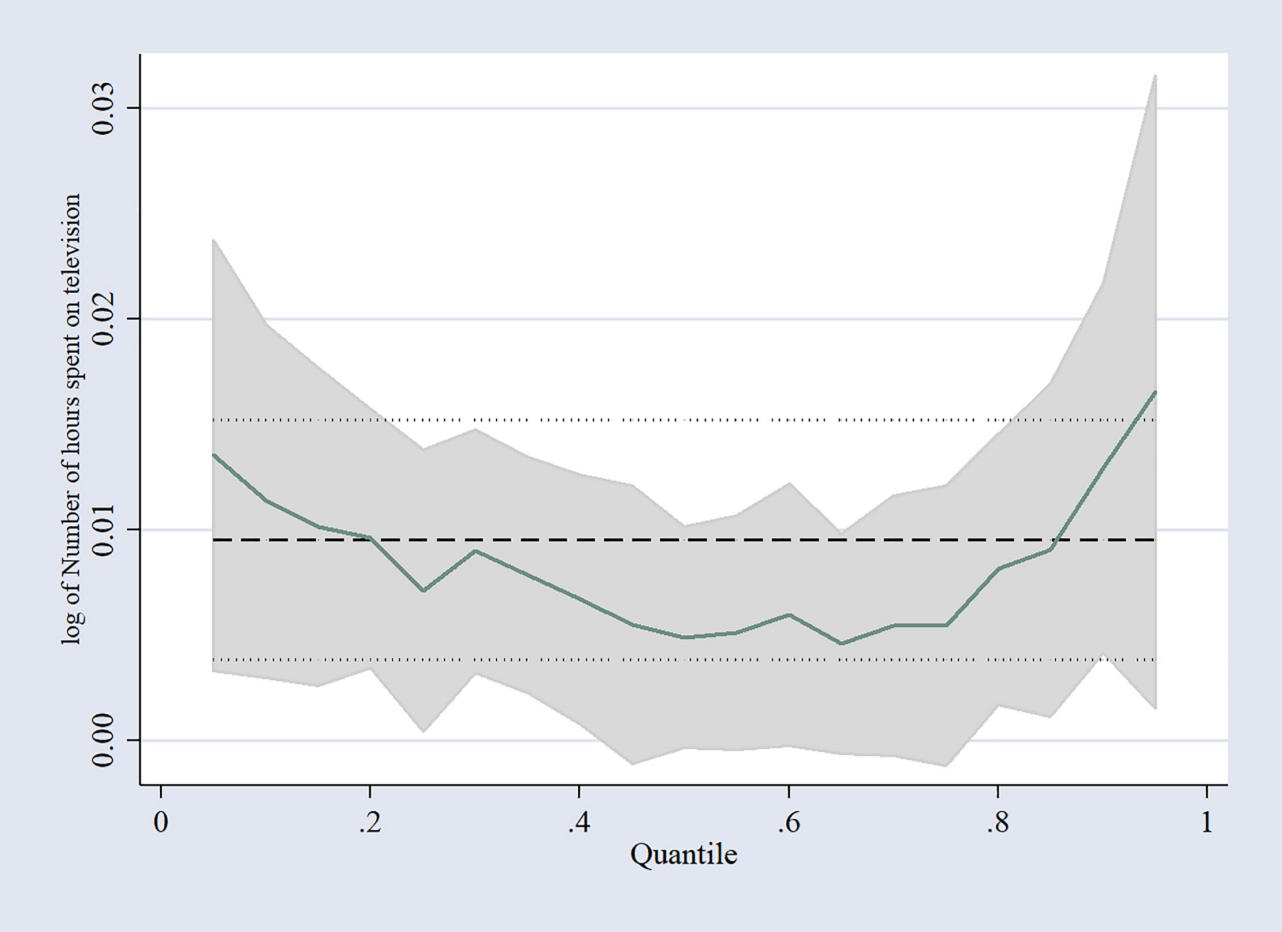
Relationship between the number of hours spent watching television and BMI across the distribution.

Since the QR coefficients in Table 3 only show the effect of the covariates at the selected quantiles, the QR coefficients were thus plotted across the whole distribution and are shown in Fig 1.

The thick line shows a plot of the various point estimates at each of the quantiles, whereas the shaded line indicates the confidence intervals. As the figure shows, the coefficient of the association of prolonged television viewing and BMI is much higher below the 20^th^ percentile and above the 90^th^ percentile, suggesting that people with BMI values within those regions of the quantiles are highly affected.

## Discussion

Using a nationally representative survey, this study is the first to investigate the association of prolonged television watching and BMI values in Saudi Arabia beyond the mean. The results show that most of the respondents had high BMI values with a mean of 28.5 (Kg/m^2^) and a median of 27 (Kg/m^2^). This implies that a large proportion of the population in the KSA is either overweight or obese. Although this is the case, the BMI values found in this study are much higher than those recorded for most of the countries in the Arab world and in the overall Asian region [44, 45].

Based on the regression, the main findings are that whilst the OLS coefficients are constant across the BMI distribution, based on the QR, this coefficient size is higher towards the lower and upper tails of the BMI and insignificant in the middle of the BMI distribution (Q50 and Q75). This indicates that prolonged television watching has a greater positive association on individuals with very low BMI values and those with very high BMI values. In other words, prolonged television watching is likely to increase the BMI values of individuals who are obese faster than those with moderate BMI values. This poses a challenge as it is the individuals who have very high BMI values who are at higher risk of suffering from non-communicable diseases, such as cardiovascular diseases [46, 47]. However, the main message is that a positive relationship between the number of hours spent watching television and BMI still holds but is heterogenous across the distribution.

The study also found a significant association between gender and BMI. The results indicate that males are likely to have significantly lower BMI values across the distribution. This may perhaps be as a result of the differences in metabolic rates between males and females. Males have higher metabolic rates such that their bodies are better able to process and utilize the calories they consume leading to lower body mass values [48]. It is, therefore, imperative that ladies take more proactive steps to watch and regulate their caloric intake and do more physical exercise. This BMI gap warrants further investigation to understand the drivers of the observed difference.

The findings that a positive relationship exists between the number of hours spent watching television and BMI are consistent with several other studies that found that prolonged television watching led to higher BMI values [49–52]. However, our results differ in the sense that the quantile regression showed no significant effect within the middle of the BMI quantile.

The positive relationship found between prolonged television watching and BMI may potentially be explained by several factors. First, the level of physical exercise seems to be lower among the Saudi population. As observed in the data, most people indicated that they do not partake in any physical exercise. This comes as no surprise given that there has been an increase in sedentary lifestyles and physical inactivity in the KSA in recent times [53–58]. This can indeed be problematic, because physical exercise has been shown to lead to lower BMI values [59, 60]. Second, as indicated by previous researchers, longer time spent watching television is associated with increased calorie consumption while watching as a result of advertising and also a reduced resting metabolism [49, 61]. Moreover, the level of soda consumption was also reported to be higher, thereby suggesting that the dietary pattern in terms of soda consumption greatly contributes to the escalating BMI problem.

This study is not without its limitations. The study shows that BMI is associated with prolonged television watching but does not take into account the problems that may arise due to self-selection into watching television. This may potentially cause endogeneity. As such, the interpretations should not be interpreted as causal [43, 62]. Second, there might be other factors beyond what we have controlled for which potentially influence the relationship of prolonged television watching and BMI. These limitations provide an avenue for further research into the relationship by taking into account the unobserved factors.

## Conclusions

This paper set out to understand the relationship between the number of hours spent watching television and BMI. BMI values were calculated as weight in kilograms divided by the square of height in meters. In order to assess the relationship across the whole distribution, the study used a continuous measure of BMI. The study used data from the SHIS, which collected data from a nationally representative sample of the KSA population and performed both descriptive and econometric analyses.

The findings suggest that watching too much television may be associated with having a large BMI. The findings also show that there is a gender gap in the BMI levels between males and females, where females have higher BMI values than males. Of interest, prolonged watching television has a much greater association at the lower and upper distribution levels of the BMI. Based on the findings, there are critical policy implications which can be suggested. There is a need to incorporate television programs to teach people about weight loss methods. This should not be exclusive to the homes only but should also include communal programs. It is also recommended that effort should be made to create recreational programs that act as alternatives to watching television so as to help the population reduce the amount of time spent watching television. Programs that require active participation would help to reduce BMI and hence levels of overweight as well as obesity. Furthermore, since BMI was found to be likely higher for females, gender specific interventions such as women sports days and females’ sports clubs could help to reduce the BMI problem.
